# Inferring transcript phylogenies

**DOI:** 10.1186/1471-2105-13-S9-S1

**Published:** 2012-06-11

**Authors:** Yann Christinat, Bernard ME Moret

**Affiliations:** 1 Laboratory of Computational Biology and Bioinformatics, EPFL, 1015 Lausanne, Switzerland

## Abstract

Alternative splicing, an unknown mechanism 20 years ago, is now recognized as a major mechanism for proteome and transcriptome diversity, particularly in mammals--some researchers conjecture that up to 90% of human genes are alternatively spliced. Despite much research on exon and intron evolution, little is known about the evolution of transcripts.

In this paper, we present a model of transcript evolution and an associated algorithm to reconstruct transcript phylogenies. The evolution of the gene structure--exons and introns--is used as basis for the reconstruction of transcript phylogenies. We apply our model and reconstruction algorithm on two well-studied genes, MAG and PAX6, obtaining results consistent with current knowledge and thereby providing evidence that a phylogenetic analysis of transcripts is feasible and likely to be informative.

## Introduction

Alternative splicing is a mechanism to produce different proteins from the same gene--the end of the paradigm "one gene, one protein." In many genomes, several, or even most, genes are split into pieces called exons, separated by regions called introns, and a splicing mechanism takes the transcribed string of exons and introns, removes the introns, and splices the exons to form a single continuous string that is then translated into a protein. In alternative splicing, a mechanism underestimated until 1990, the splicing produces variants in which some of the exons can be omitted (and occasionally even some of the introns retained), thereby causing different proteins to be produced. Alternative splicing exists to some degree in most eukaryotes, but is most frequent in the more complex lineages. Thus it is present, but limited, in plants and fungi, but quite common in vertebrates--some researchers have conjectured that up to 90% of the human genes are alternatively spliced [1-3]. Alternative splicing is now recognized as a major mechanism for proteome and transcriptome diversity [4,5].

The implications of this shift in paradigm are significant. The basic model of transcriptome evolution--DNA modification at the gene level and alternative transcription start sites--is incomplete: any modification that affects the splicing mechanism has to be considered. However, while evolution of DNA at the gene level has been the subject of intense scrutiny for decades, very little is known regarding the changes in the splicing products of alternative splicing. Thus there is a need to define a model of evolution for transcripts, not at the nucleotide level, but at the splicing level--which exons (and introns) are included, which excluded?

A better understanding of the relationships among different transcripts would benefit annotation transfer. Different proteins from one gene may have different functions, may be localized to certain tissues, or may be present at different developmental stages. Knowledge of their evolution would help in assessing the function of their homologues. Transcript phylogenies would also contribute to next-gen sequencing methods, especially RNA-seq. For instance, the "DREAM6 Alternative Splicing Challenge" asks to reconstruct alternatively spliced mRNA transcripts from short mRNA-seq data without a reference genome, but using the transcriptomes of other organisms [6]. A transcript phylogeny would help in assessing the support value of a predicted transcript.

In this paper, we propose a model of transcript evolution and an associated algorithm to reconstruct transcript phylogenies.

## Transcript evolution

### Background

Many studies have been published on the rate of exon insertion and deletion and on the statistics of different types of splicing, but few researchers so far have studied the evolution of transcripts [2]. Harr and Turner showed that most transcripts among *Mus *subspecies were novel [7]. Nurtdinov *et al*. compared the human and mouse transcriptomes and concluded that half of the genes give rise to species-specific isoforms and only three quarters of all isoforms are present in their orthologous genes [8]. Splicing is also affected by non-DNA events. Modification of the chromatin structure can yield changes in the expression of a given transcript and may even create a new transcript or silence an existing one [1]. Finally, a few groups studied the correlation between gene duplication and alternative splicing [9-11].

These studies indicate that alternative splicing is a fast-evolving mechanism and hint that most of the transcripts may be little more than evolutionary noise. These studies also indicate that groups of species share a significant number of transcripts, whose relationship can only be delineated with a more complete model.

### A model of transcript evolution

In the description of alternative splicing, the simplest concepts are those of *constitutive *exons, which are part of every transcript, and of *cassette *exons, which may or may not be present in any given transcript. In general, any exon that is not constitutive is called a*lternative*. There exists other types of splicing mechanisms, of which alternative 3'- or 5'-sites and intron retention are the most frequently cited [1,3,5,12,13]. Note that the definition of a constitutive exon requires that all transcripts for a given gene be known. If, however, alternative splicing is closer to a biased random sampling from the space of all possible isoforms (so that every possible splice form is produced at some or other time), then there may be no such thing as a constitutive exon. As the debate on this issue continues and as our aim is to provide a model against which to test various hypotheses regarding transcript evolution, we develop a model in which we consider the existence of constitutive exons as a given.

We thus model a transcript as a subset of the gene exons. We model alternative 3'- or 5'-sites as constitutive exons with two or more internal states--each state encoding for one particular configuration. Finally, we assimilate intron retention to cassette exons. We model transcript evolution as a two-level process. The gene structure, viewed in terms of its collection of exons and introns, constitutes one level, while the collection of transcripts obtained from that structure constitutes the other level. Modification of the gene structure affects the transcriptome, but modification of the transcriptome does not affect the gene structure. Peng and Li [14] showed that the status of an exon, constitutive or alternative, is conserved through tandem exon duplication, a finding that hints at a model of evolution where the status of an exon is encoded at the gene level. Consequently we have three possible exon states in our model of gene evolution: absent, constitutive, or alternative. We assume that all transitions--birth, death, and mutation between constitutive and alternative--are equally likely.

In addition to the model of exon evolution at the gene level, transcripts can gain or lose exons. Table 1 sums up the possible evolutionary changes at the transcript level, given the evolution of a particular gene exon. Note that a transition from alternative to alternative does not imply that the exon will still belong to the same transcripts.

**Table 1:** Evolutionary events on the transcriptome


↗	0
0	No transcript had this exon and none will have it.
1*_A_*	Some transcripts had this exon and none will have it.
1*_C_*	All transcripts had this exon and none will have it

↗	1*_A_*
0	No transcript had this exon and some will have it.
1*_A_*	Some transcripts had this exon and some will have it.
1*_C_*	All transcripts had this exon and some will have it.

↗	1*_C_*
0	No transcript had this exon and all will have it.
1*_A_*	Some transcripts had this exon and all will have it.
1*_C_*	All transcripts had this exon and all will have it.

All evolutionary changes for transcripts for a given exon at the gene level. 1*c*: constitutive exon 1*_A_*: alternative exon, 0: no exon

Finally we assume that a transcript can undergo a lethal mutation or be subject to regulation and disappear at any time. In a manner similar to gene duplication, new transcripts may also be created at any time during evolution.

**Figure 1:**
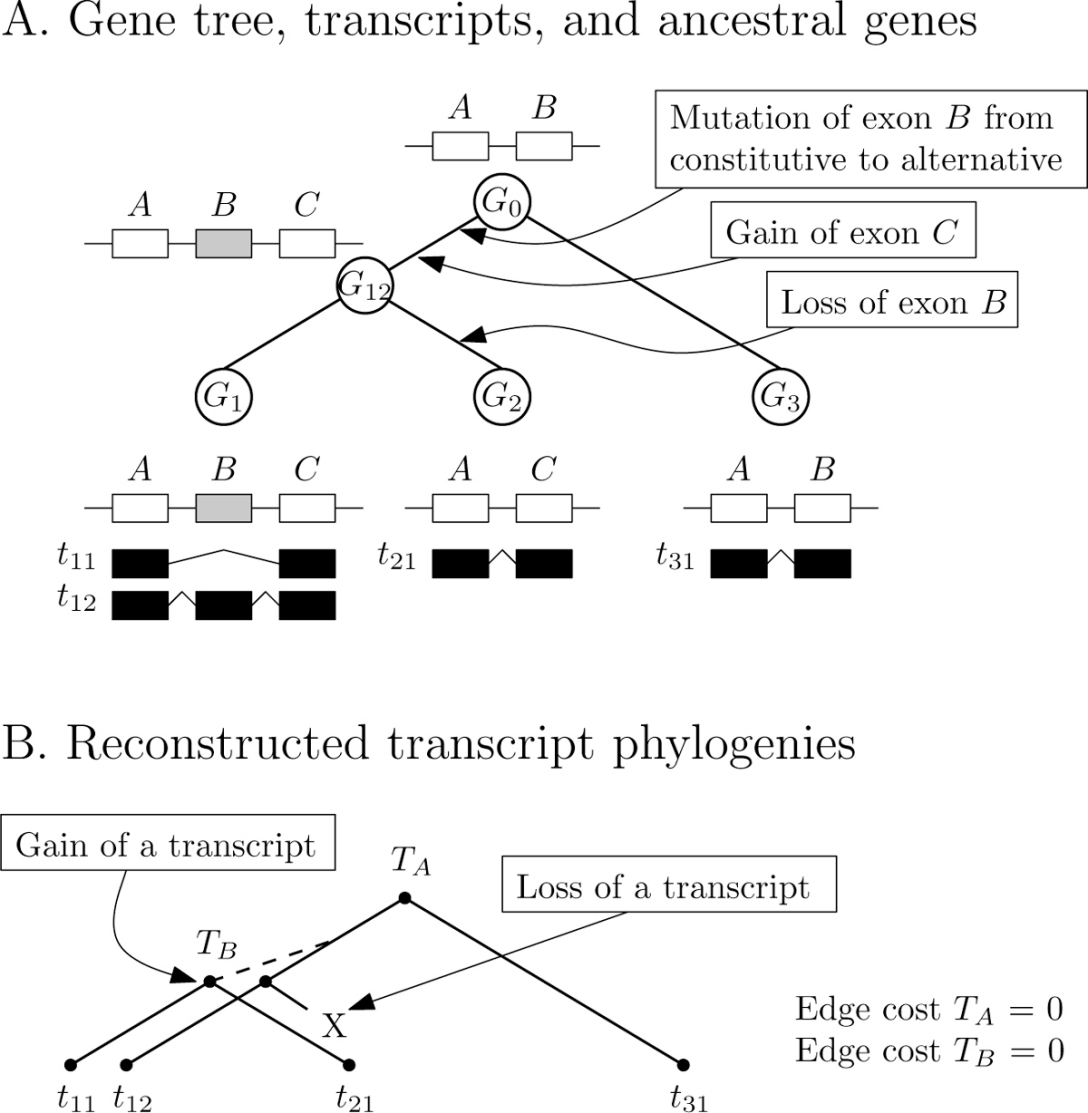
**Illustration of the two-level model**. The first level is represented in *A *where the gene evolution happens. In *B*, one can see the transcript phylogeny. The total edge costs of *T_A _*and *T_B_* are both zero. While this is expected for *T_B _*as transcripts *t*_11_ and *t*_21_ share the same exons, it is not obvious for *T_A_*. Transcripts *t*_12_ and *t*_31_ differ by exon *C*. However the latter was gained during the evolution from *G*_0_ to *G*_12_. This event belong to the first level and thus has zero cost in the transcript phylogeny. The dotted line represents the hidden relationship of the new transcript to its ancestor. In the extended model, that link would be revealed.

The model focuses on transcript evolution and the cost reflects only transcript events. Any gene-related evolutionary event--gene duplication and loss, exon gain and loss--has zero cost. For instance, the gain of a constitutive exon in the gene will automatically affect all transcripts and thus will not be reflected in the total cost. This concept is illustrated through an example in Figure 1.

Our model yields a forest of *transcript trees*, which represents the evolution from ancestral transcripts to observed transcripts. Each transcript tree is a subtree of the gene tree, since all transcripts arise from that gene family and, if they evolve, must evolve on the same tree. If a new transcript arises from an existing one, the new transcript will be considered as the root of a new transcript tree. Our basic model uses a fixed cost for the creation of new transcripts. Of course, the basic model does not assume that transcripts are created *ab initio*; rather, it postulates a hidden relationship with an unknown ancestor.

New transcripts arise from existing ones and thus are the result of evolutionary changes that may legitimately correspond to different costs. We use a fixed cost for simplicity and also because it leads to a very efficient pruning of the search space. We have designed and implemented an extended model in which the creation of a new transcript is dynamically assigned a cost that corresponds to its evolution from its closest ancestor (a maximum parsimony approach). However, the dynamic cost computation prevents good pruning and makes the problem intractable for medium-sized instances.

Our algorithm starts by reconstructing the exon structure of the ancestral genes, then looks for the most parsimonious forest of transcript trees. For the ancestral gene reconstruction algorithm, we used a maximum parsimony approach, using Dollo's parsimony--that is, an exon cannot be created twice [15,16].

## Results

The algorithm was tested on two well studied gene families to assess the correctness of the model on biological data. Further testing was done on simulated data to test the algorithm itself.

### Results on the MAG gene

The Myelin-Associated Glycoprotein (MAG) is a neuronal transmembrane glycoprotein that acts both as a ligand for an axonal receptor and as a receptor for an axonal signal [17]. It has been extensively studied and due to its short length and limited alternative splicing, it makes a perfect candidate for testing our algorithm.

**Figure 2:**
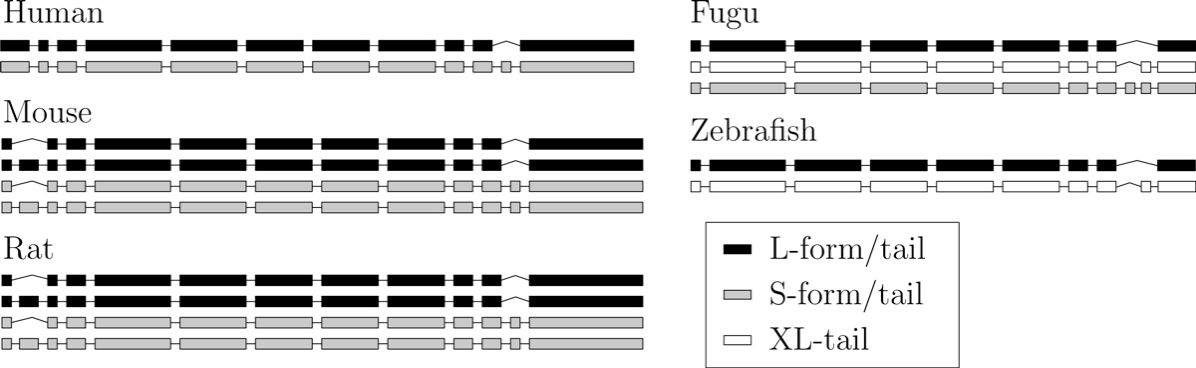
**Transcripts of the MAG gene family**. Transcripts are color-coded by their isoforms.

Two main isoforms are known in mammals: L-MAG and S-MAG. The S-MAG is created by the inclusion of the penultimate exon that creates an early stop codon and hence removes the cytoplasmic domain. In rodents the second exon is also alternatively spliced and occurs in both the S- and L- forms, yielding four transcripts in total [18]. Two major MAG isoforms have been observed in both zebrafish and fugu: L-tail (exon 9, from the left, is skipped) and XL-tail. The retention of the eighth intron in the fugu fish yields a third form (S-tail), which is not observed in the zebrafish [19]. The transcripts corresponding to these isoforms are displayed in Figure 2.

#### Data

**Figure 3:**
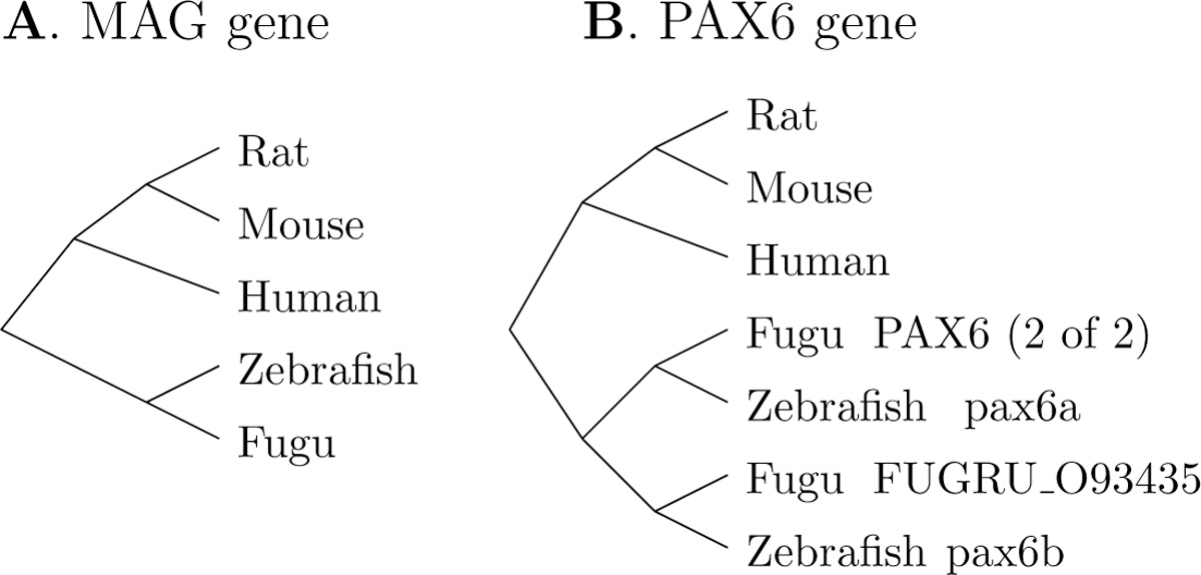
**Gene trees**. Gene trees for the MAG and the PAX6 gene families across five species.

Transcripts and exons for the five species were compiled from the literature [17-21]. The sequences and the gene tree, as shown in Figure 3-A, were obtained from the Ensembl database [22].

**Figure 4:**
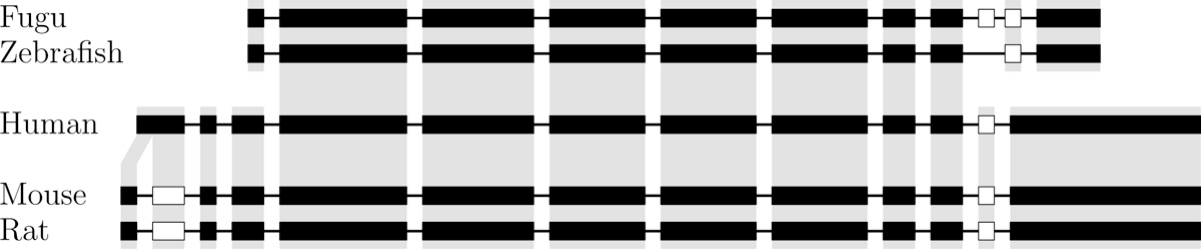
**Orthologous exons of the MAG gene family**. A gray background indicates orthologous exons. Constitutive exons are shown in black. Note that alternative exons are not conserved between mammals and fishes.

We concatenated the gene's exons and aligned the resulting sequences using Mauve [23]. Every exon either was not aligned to any other exon or provided close to one hundred percent coverage of its ortholog. The only exception was the first human exon, which corresponds to the first two exons of the rodents. Such a situation might have posed a problem had the human exon been alternative, but fortunately it is a constitutive exon. The first human exon was consequently modeled as two exons. The eighth intron of the fugu fish, which triggers an early stop codon, could not be aligned to any exon in any other species. Orthologous exons were then inferred from this alignment and are shown in Figure 4.

#### Results

**Figure 5:**
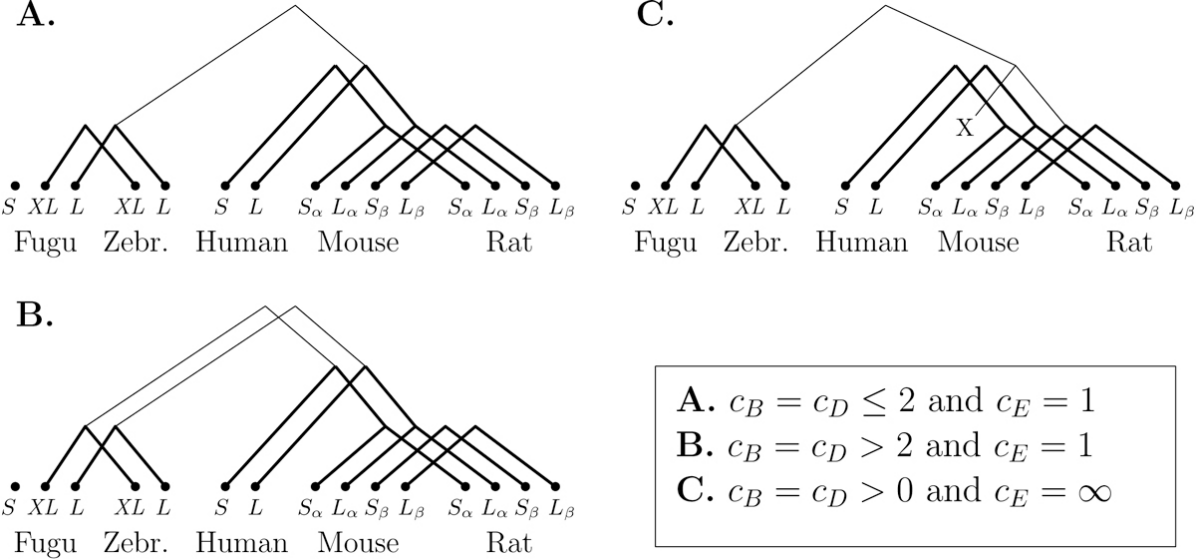
**Transcript phylogenies for MAG under the basic model**. Inferred transcript phylogenies for the MAG gene under the basic model and different costs. Transcripts *S_β _*and *L_β _*in rodents represent the S- and L-forms in which exon 2 is skipped. Thicker trees contain similar transcripts. Only solutions with a minimal number of events are displayed.

We tested two setups. In the first experiment, we used a cost of infinity for exon gain/loss, *c_E_*= ∞, whereas in the second we used a unit cost, *c_E_*= 1. An infinity cost for exon gain/loss allows us to test if some transcripts are exactly similar in the second level of the model. The cost of every transcript tree is consequently either zero or infinity. Note that two transcripts with different exons can be reunited under a zero-cost tree as exon gains and losses at the gene level may explain the difference. In both setups, the cost of transcript birth, *c_B_*, and death, *c_D_*, was set to a single parameter and varied. As shown in Figure 5, each experiment yielded solutions consistent with our biological knowledge of the isoforms. The S- and L-forms in mammals and the L- and XL-tail in fishes are clustered on their respective trees. However, the relationship between the fish and mammal isoforms is unclear. If the cost of exon gain is infinity, then the only relation between fishes and mammals is a link from the L-tail to the alternative L-form in rodents--but our model requires such a link, since it demands that all genes be connected. The same reasoning applies for *c_B_*= *c_D_*≤ 2 and *c_E_*= 1. The cost of connecting a mammal transcript to a fish transcript is always greater than the cost of adding a new tree. The S-tail in the fugu fish is isolated and shows no relation to the S-form of mammals. Its distance to the mammal S-form is too great to allow the two to be clustered. There is no evidence that those two transcripts are biologically related, nor do their sequences align well--their only common feature is that both induce an early stop codon.

#### Results with the extended model

**Figure 6:**
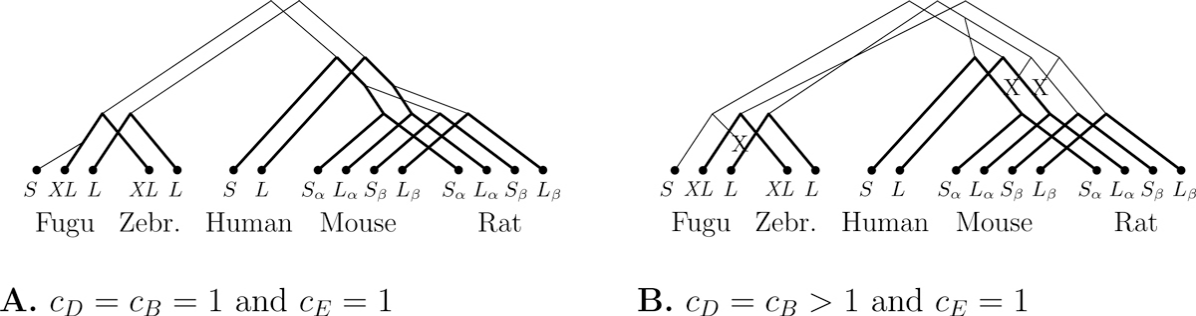
**Transcript phylogenies for MAG under the extended model**. Inferred transcript phylogenies for the MAG gene under the extended model and different costs. Note that we have 3 transcript losses for *c_B_*= *c_D_*> 1.

Since the search space for the MAG instance is small, we were able to run the extended model on it. As seen in Figure 6, the result on *c_B_*= *c_D_*= 1 is the same as for the basic model using *c_B_*= *c_D_*> 2, except that newly created transcripts are linked to their closest ancestor. For *c_B_*= *c_D_*> 1, our algorithm reconstructed three ancestral transcripts. Isoforms are still well clustered within fishes and mammals but the relationship between them seems complicated. For instance, the fugu S-form is linked to the "standard" L-form in mammals, which seems a bit unlikely. When *c_E_*= ∞, no phylogeny could be found that did not have an infinity score.

Many solutions of minimum cost may exist. Consequently our algorithm can return several solutions. In order to sort the best solutions, we computed the total number of events including exon gain and loss at the gene level. This process acts as a second filter. However, the number of solutions is highly informative. For instance, Figure 6-B shows only one of the 32 solutions whereas only 2 solutions were found for *c_D_*= *c_B_*= 1. This indicates that the phylogenies for *c_B_*= *c_D_*> 1 are far from being certain and should thus be considered with extreme care. Moreover, only constitutive exons are shared between fishes and mammals. Consequently, the edges linking a fish transcript to a mammal transcript will highly depend on the ancestral gene reconstruction.

### Results on the PAX6 gene

The PAX6 gene is part of the well-studied paired box gene family (PAX), which encodes transcription factors for many developmental processes and is subject to heavy alternative splicing [24-26]--41 transcripts were found in a gene in the pigeon retina [27]. The canonical isoform is characterized by an N-terminal paired domain followed by a linker, a paired-type homeodomain, and a (P/S/T)-rich C-terminal domain, yielding a 422-amino-acid protein (437 in zebrafish). The gene undergoes alternative splicing and the best-known alternative isoform, +5a, differs from the canonical isoform by the inclusion of exon 5a. This 14-amino-acid insertion in the paired domain disrupts the DNA-binding ability of the N-terminal domain and enhances the binding of the C-terminal domain, thus creating a new set of interactions [28]. As can be seen in Figure 3-B, gene duplication occurred in the fish species leading to two PAX6 genes in the zebrafish and the fugu fish.

#### Transcripts and orthologous exons

Mammal transcripts were obtained from the Human-transcriptome Database for Alternative Splicing (H-DBAS) [29] and fish transcripts from the Ensembl database [22]. In the H-DBAS database, we considered only transcripts that were present in both the cDNA and mRNA databases, except in the case of *R*. *norvegicus*, where only the mRNA database was available. Similarly, with the Ensembl database, we used only transcripts that had CDS or UTR support. The gene tree was obtained from the Ensembl database and genes are thus named accordingly. The canonical and the +5a transcripts were identified through their protein product and the literature.

The literature on the PAX6 gene in the fugu fish is very sparse--we could find only one article, by Miles *et al*. [30], but that article does not corroborate the information in the Ensembl database. Thus we used the Ensembl data, as it is more recent, but we have no ground truth regarding the canonical or alternative isoforms.

**Figure 7:**
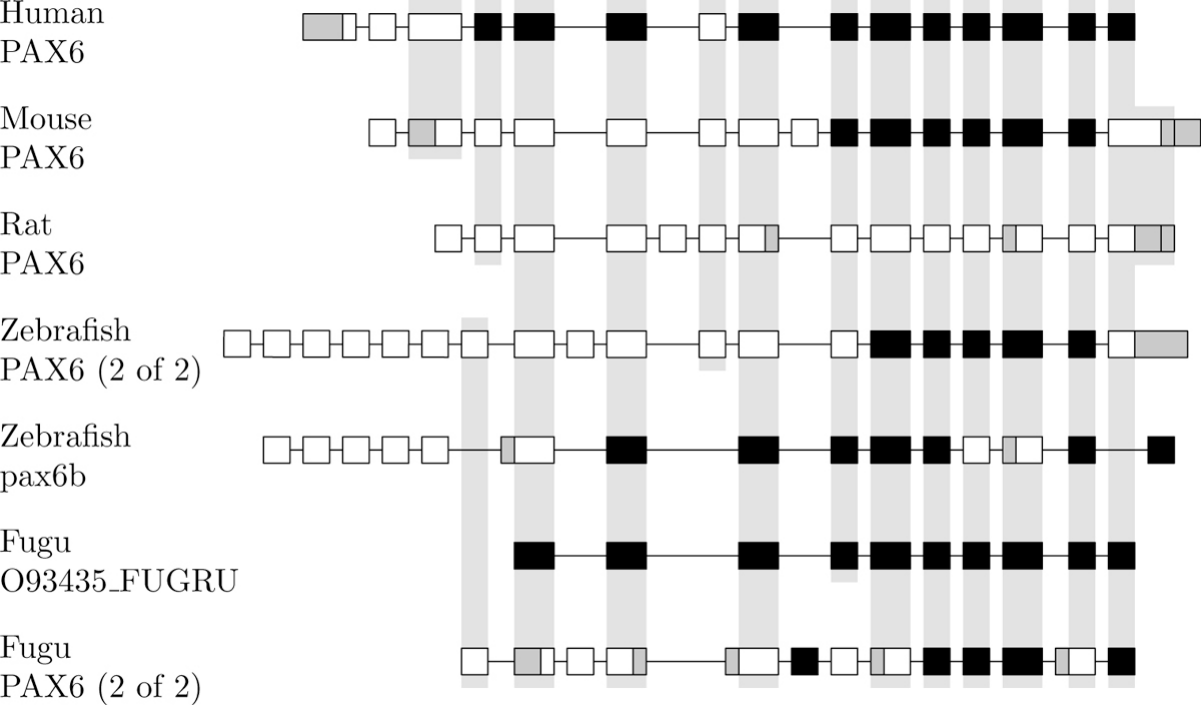
**Orthologous exons of the PAX6 gene family**. Orthologous exons of the PAX6 gene for the 7 genes of the 5 species. A gray background indicates orthologous exons. Constitutive exons are shown in black. Alternative 3'- and 5'-end are shown in gray. Note that only exons belonging to a transcript are shown.

**Figure 8:**
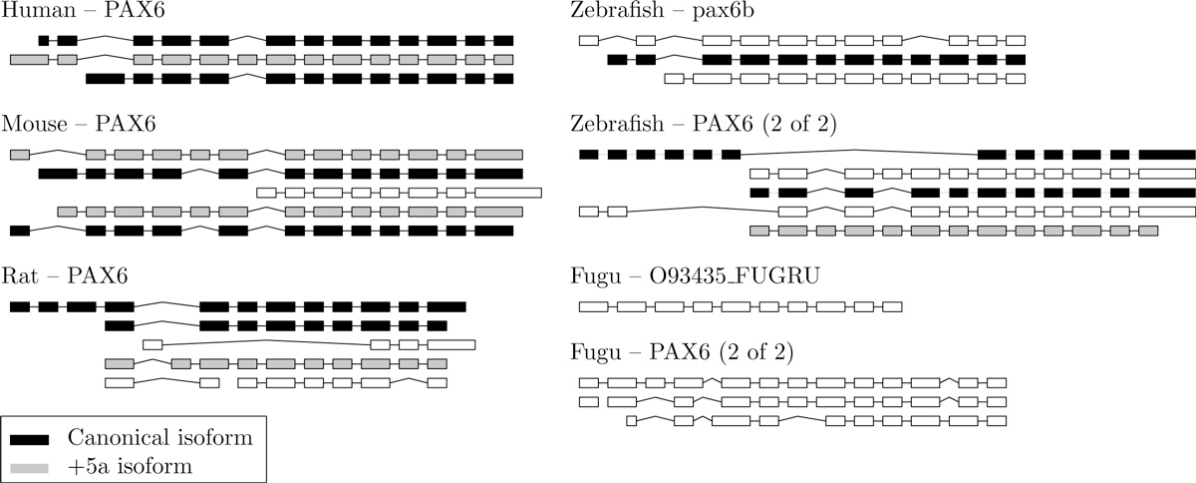
**Transcripts for the PAX6 genes**. The canonical isoforms are shown in black and the +5a isoforms in gray. Note that several transcripts are translated into the same isoform.

The H-DBAS database conveniently indicates orthologous exons for the mouse, rat, and human. We ran an all-against-all semi-global alignment of all exons to confirm the mammalian orthologs and to obtain the orthologs for the two fishes. Orthologous exons are shown in Figure 7 and transcripts in Figure 8. In all species, we observe that several transcripts can produce the same isoform.

#### Results

**Figure 9:**
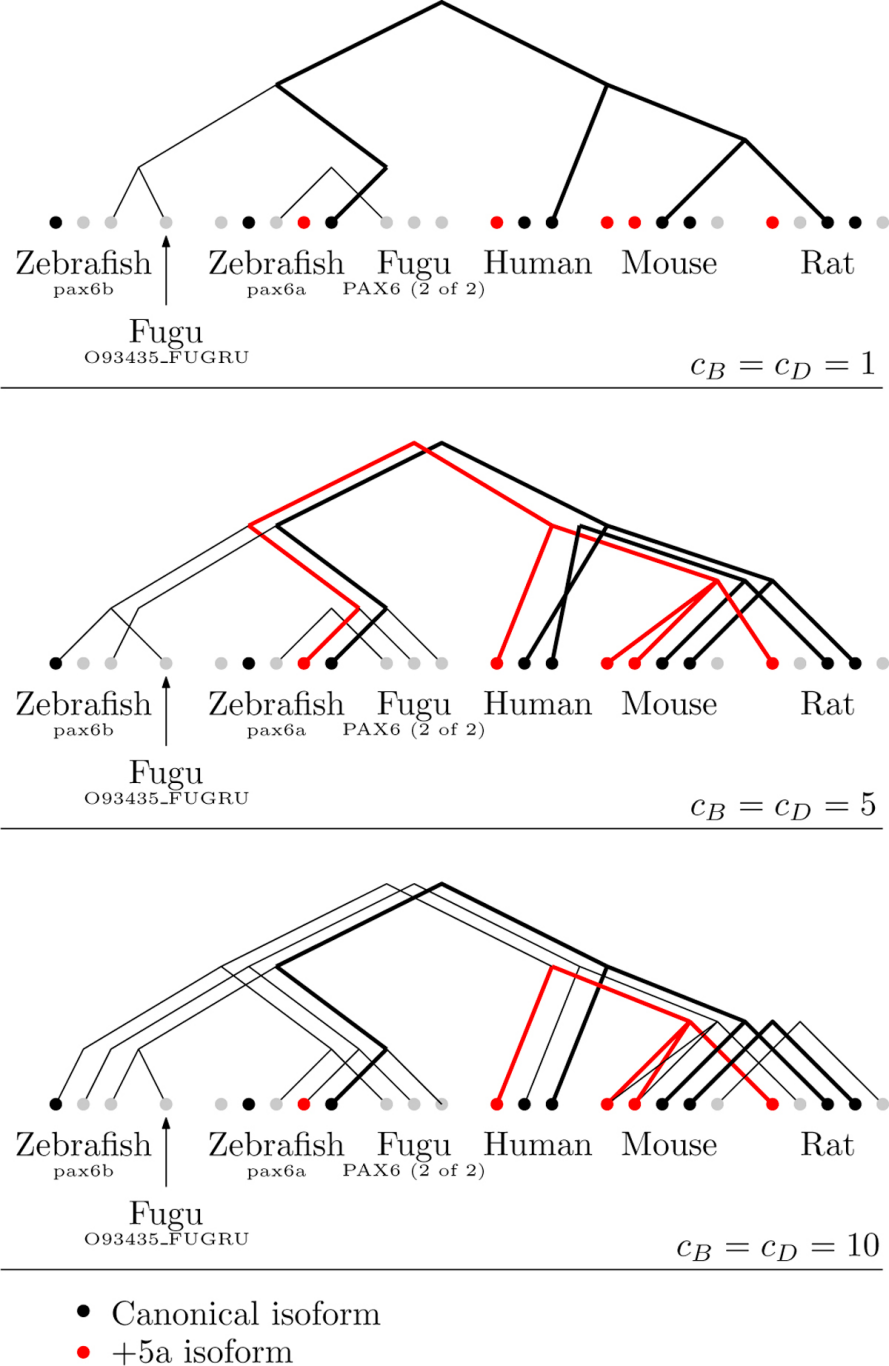
**Transcript phylogenies for the PAX6 gene family**. Transcript phylogenies for the PAX6 gene for different values of transcript birth, *c_B_*, and death, *c_D_*. Multiple solutions are superimposed. Thicker lines connect similar isoforms.

As with the MAG gene, we tested two setups, with unit and infinite costs for exon gain or loss. However no solution could be found with *c_E_*= ∞. Figure 9 reveals a correlation between *c_B_*, *c_D _*and the number of ancestral transcripts. A higher *c_B_* affects the total number of trees. Any hypothesis should thus be tested under different parameters before drawing any conclusion. The best result uses *c_B_*= *c_D_*= 5, showing well-clustered isoforms within mammals. Note that the model imposes a link between all genes, so that the relevance of a single connection between fishes and mammals at low values of *c_B_* is uncertain.

**Figure 10:**
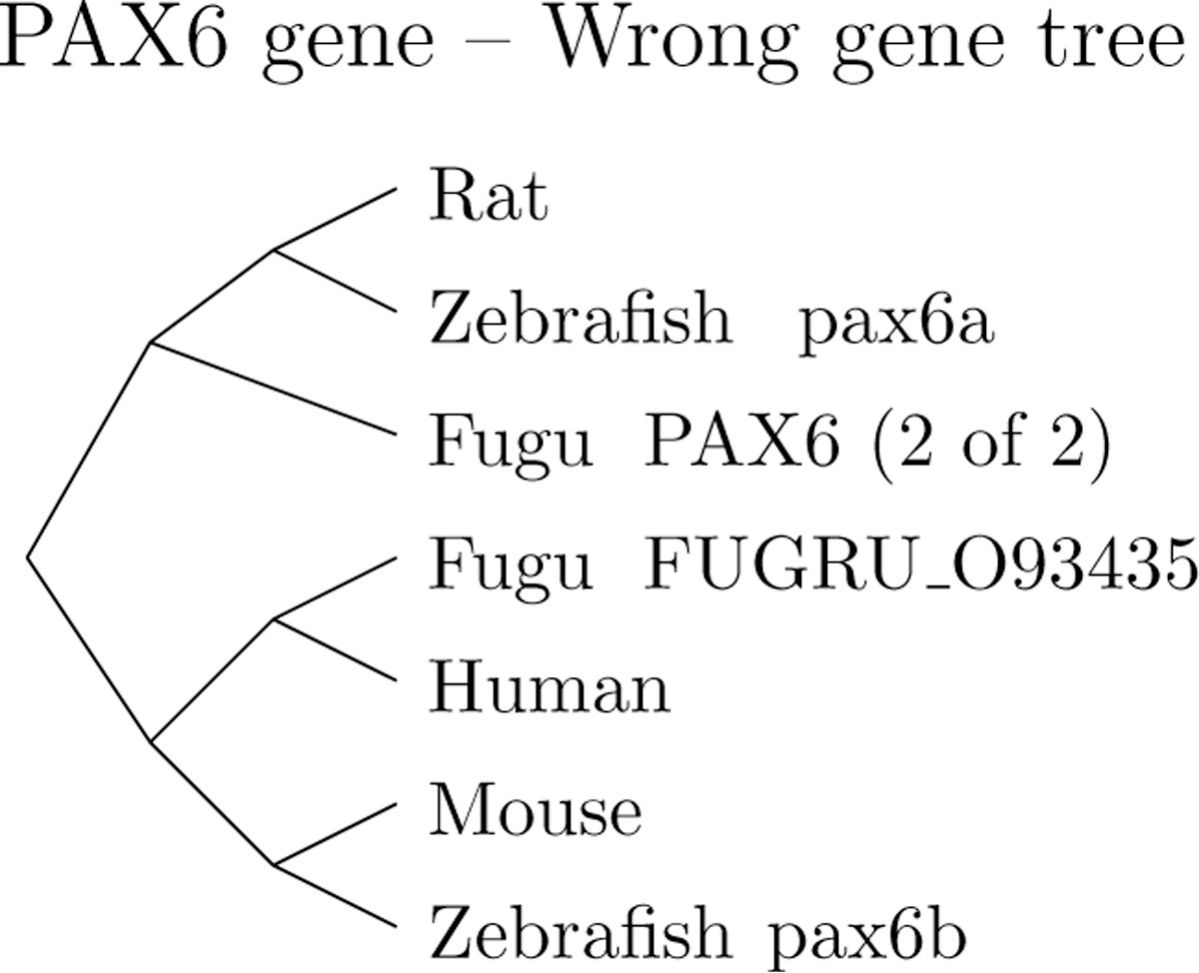
**Wrong PAX6 gene tree**. A wrong gene tree for the PAX6 family. Some leaves are shuffled.

The number of solutions with minimum cost also increases along with *c_B_*and *c_D_*. The algorithm returns 36 solutions of equal cost for *c_B_*= *c_D_*= 5. We tested the same setup under a wrong gene tree. As shown in Figure 10, we kept the structure but shuffled the leaves. Under this setup, the number of solutions increased nearly tenfold for the same parameters--a change that gives us confidence that phylogenetic information is indeed contained in the transcripts. Note that Figure 9 shows only solutions that have a minimum number of evolutionary events among solutions of minimum cost.

### Results on simulated data

In order to test the performance of the algorithm, we designed a simple scheme to generate transcripts with a given tree structure. Starting with *n_T_* ancestral transcripts at the root and *n_E_* random exons, each gene exon can either be born or die independently along the tree. The same evolutionary process applies to transcripts with exon gain or loss depending on the current set of gene exons.

**Figure 11:**
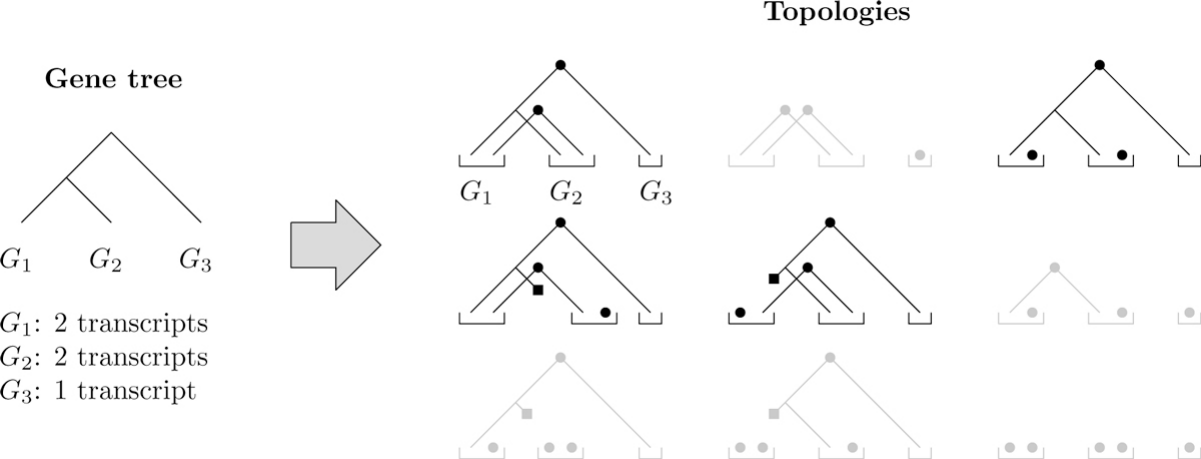
**A topology example**. The 9 topologies corresponding to a trivial 3-gene example. In a topology, a dot represents a transcript birth and a square a transcript death. Grayed out topologies are not valid as some genes are unconnected.

**Figure 12:**
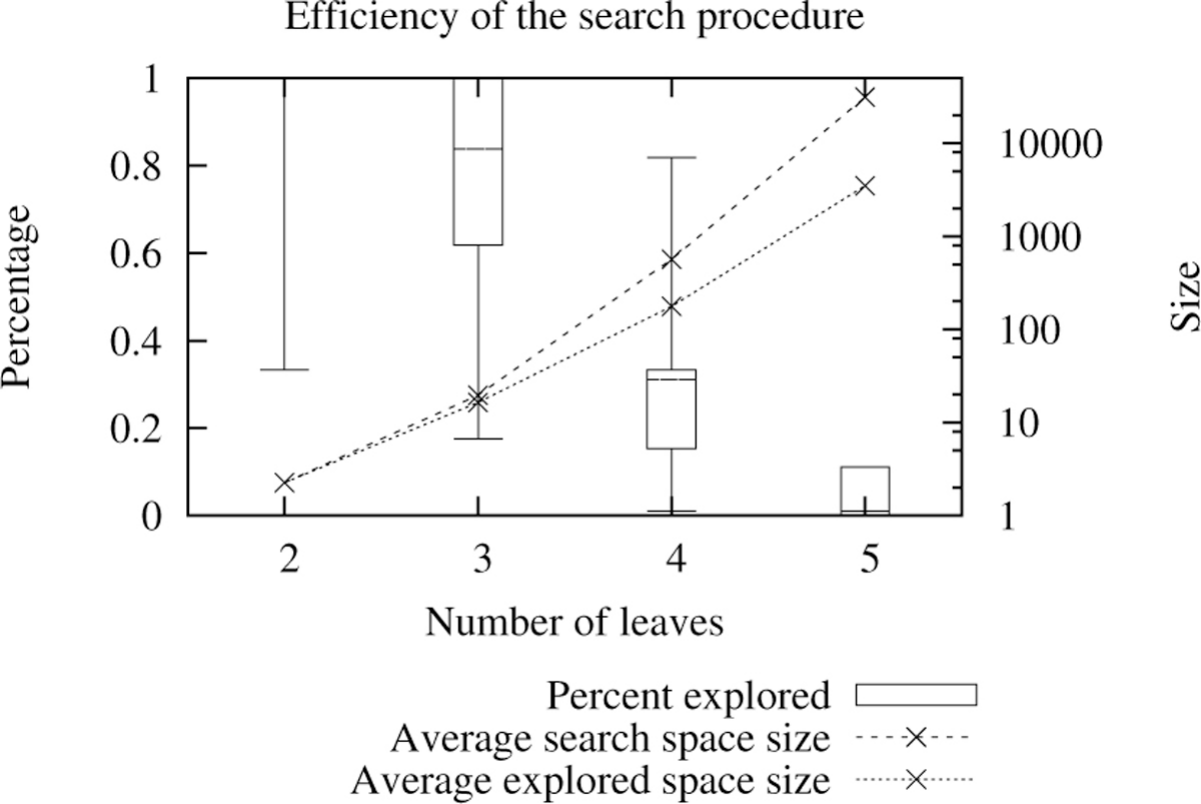
**Efficiency of the search procedure**. Percentage of topologies that get passed to the leaf assignment procedure. The average number of topologies per number of leaves is plotted on the second Y axis. Box plots and average sizes were computed on 1000 runs.

The reconstruction algorithm works by splitting the search space into topologies--a topology being a forest of transcript trees whose leaves are not assigned. Figure 11 illustrates the topology space on a simple 3-gene example. The algorithm first explores the topology space rapidly then finds the best leaf assignment on good candidates. (More details are given in the methods section.) The search on the topology space is optimal, but under unfavorable circumstances may explore the entire search space. For a given number of genes, we tested the algorithm on *caterpillar *trees (trees where one of the two children is always a leaf) with a random number of ancestral transcripts. Caterpillar trees represent difficult instances since the depth of the tree is maximized for the number of leaves. Figure 12 shows that the percentage of topologies that get passed on to the leaf assignment procedure decreases quickly as the number of leaves increases. The size of the search space grows faster than exponential, but the search procedure reduces the growth rate of the number of refined topologies. Still, the growth rate of the explored space is large but it gets closer to an exponential behavior.

**Figure 13:**
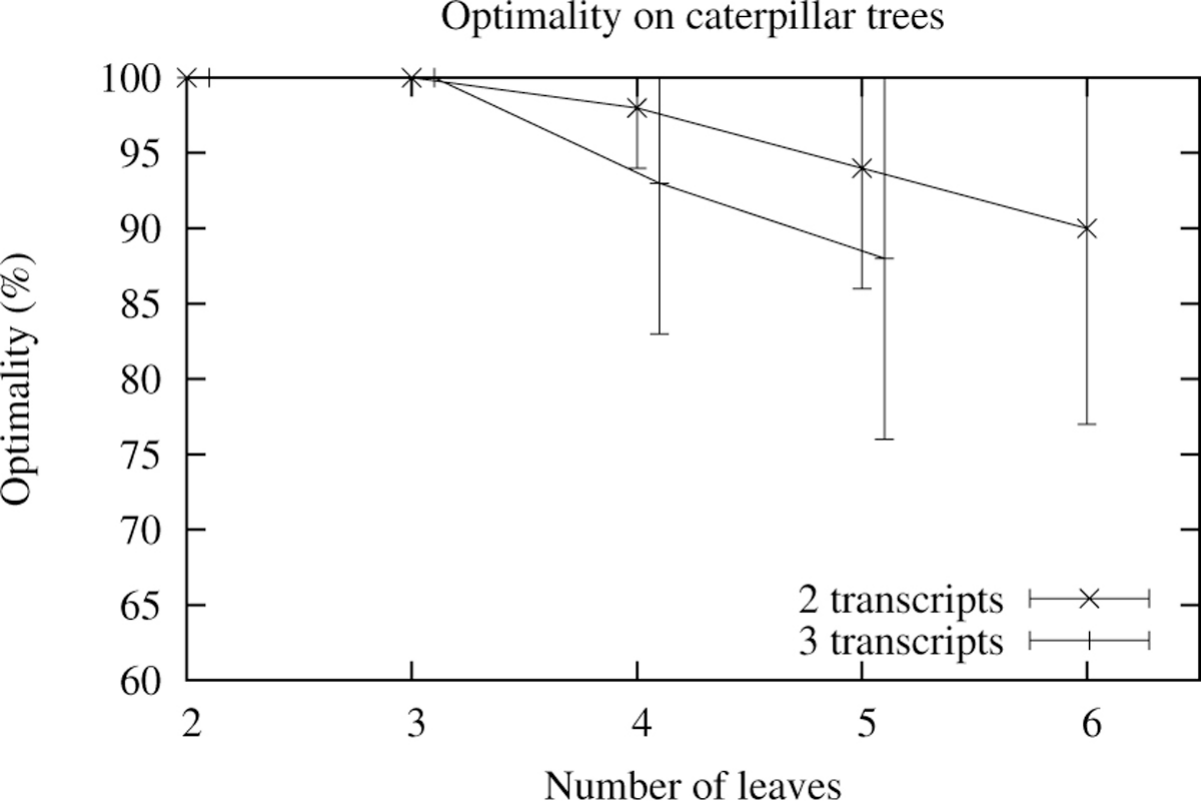
**Optimality of the leaf assignment**. Optimality of the leaf assignment algorithm for caterpillar trees. Error bars show the standard deviation on a hundred runs.

As the leaf assignment algorithm is not optimal, we tested how often it yields the best solution. Given a gene tree and its associated transcripts, for every topology, every possible transcript assignment is generated. The best score is retained and tested against the solution proposed by the leaf assignment algorithm. We define optimality as the percentage of occurrences where the leaf assignment algorithm yields the same score as the optimal solution during a single run. We performed one hundred runs, each with randomly generated hundred-exon genes, on difficult trees (caterpillar trees) and tested the optimality for different numbers of genes and of transcripts per gene. (The sizes of the instances are necessarily limited by the exhaustive search.) Figure 13 shows that, as expected, the optimality decreases as the number of leaves or transcripts increases. The large deviations come from the simulation process that allows random transcript birth and loss. Two instances may thus differ quite significantly even though they share the same number of ancestral transcripts.

**Figure 14:**
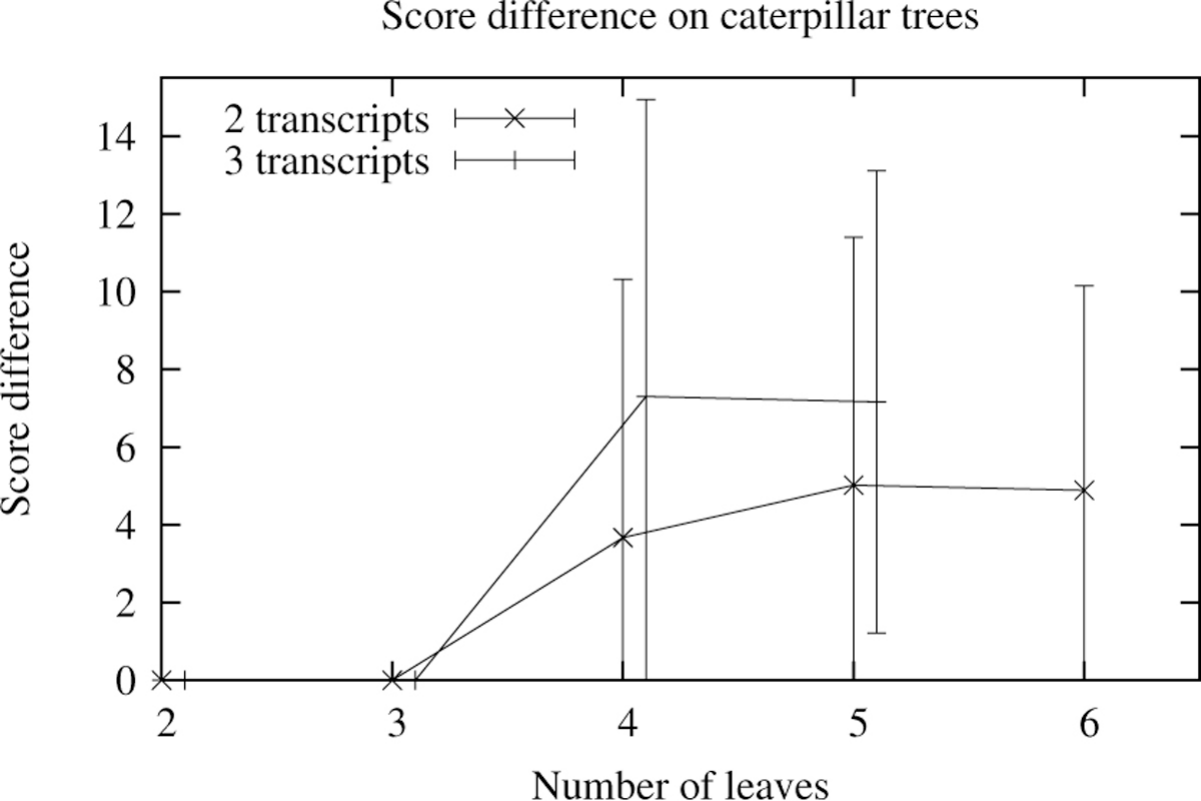
**Difference to the optimal score**. Average difference between the optimal and the reconstructed score per topology. Error bars show the standard deviation on a hundred runs. Values seem to stabilize to a constant.

The optimality criterion, as we described it, is rather strict. It fits well for small instances and allows tracking of programming errors. (For instance a tree with one transcript should always return the minimum score.) However as the trees grow larger, the optimality criterion will indicate that most solutions are not optimal but will not give any information on the badness of the non-optimal solutions. Consequently, we looked at the difference between the optimal and reconstructed score for each topology. As shown in Figure 14, the results do not look as bad as with the optimality criterion. The difference is indeed increasing but in a logarithmic fashion and seems to stabilize to a constant. As the number of transcripts and leaves increases, few solutions will have the optimal score but they will not be far from it. Note that up to three leaves or if there is only one transcript, the algorithm always returns the optimal solution. This is due to the algorithm which, by design, performs an exhaustive search for any tree of depth two or less.

We also tested the algorithm for scalability, since the running time grows faster than exponential in the worst case. Running the algorithm on the MAG gene takes a few seconds while running it on the PAX6 gene takes a few days. However our focus in this paper is to validate the concept of transcript phylogenies and show that reconstruction, within some limits, is possible. Past this step, we are confident that a heuristic can be designed to handle large problems with reasonable accuracy.

## Methods

The input of the algorithm is a gene tree with a set of leaf transcripts and orthologous exons. (Paralogous exons are considered as unrelated.)

The algorithm begins by reconstructing the state of the ancestral genes' exons--absent, alternative, or constitutive--using Sankoff's algorithm for the small parsimony problem [31]. Without any further knowledge on exon evolution, we assumed that every transition has equal cost. A constraint is added to the algorithm to ensure that the result is consistent with Dollo parsimony--that an exon cannot be created more than once.

Transcript phylogenies are then reconstructed using a two-step algorithm. For each topology, a lower bound is computed. If the lower bound is higher than the minimum encountered so far, then the topology is discarded, since there could not exist a solution with this topology with a lower score than the current optimum. Otherwise the best solution for this topology is computed. We call this last step the *leaf assignment *step; it is the only part of the algorithm that makes use of the previously reconstructed evolution of the gene's exons.

Now, in order to prune the search space efficiently, we need to establish quickly a rather good solution. Since the algorithm explores the topology space in a deterministic, breadth-first search manner, it could, in the worst case, move from worst to best topology, improving the score at each step, and thus unable throughout the procedure to prune any part of the solution space. To make such a behavior extremely unlikely on any data, we establish initial solutions by randomly sampling the search space for each number of trees before the exploration of the search space starts and retaining the configuration with the lowest score as an upper bound.

**Figure 15:**
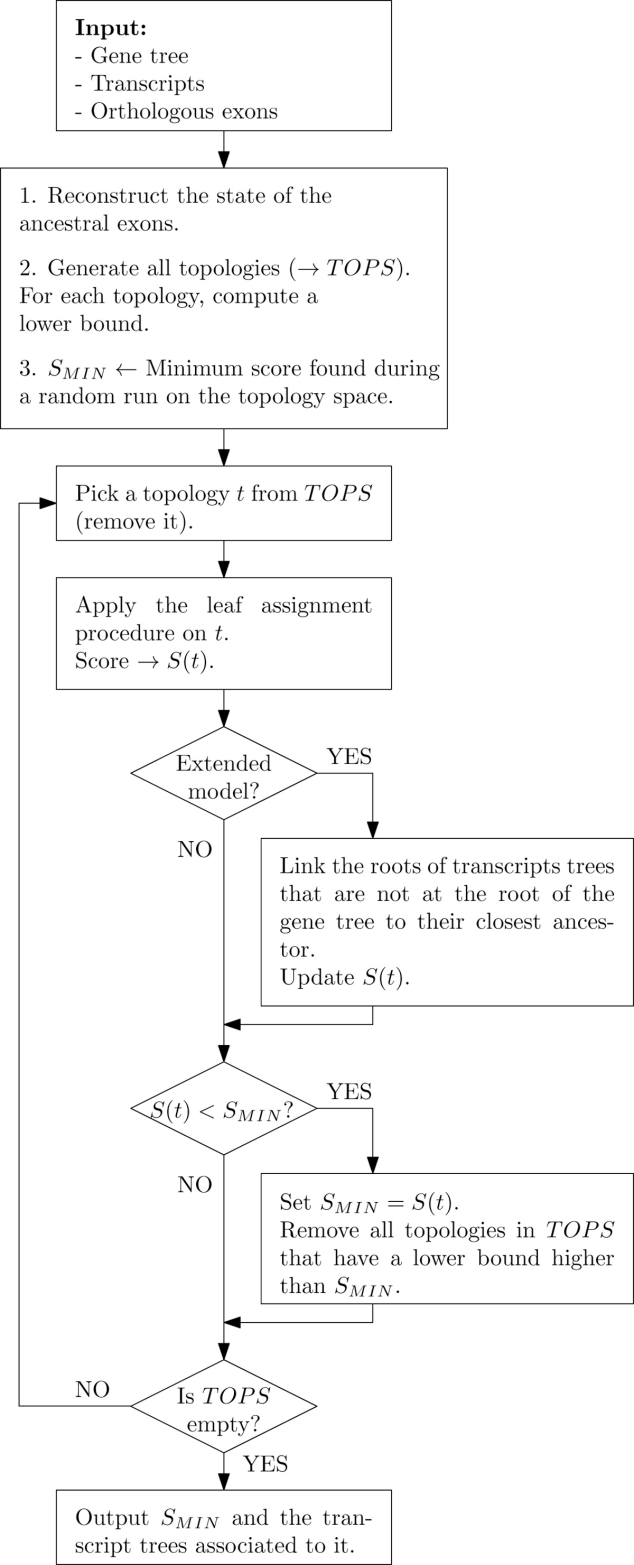
**Main algorithm**. Sketch of the main algorithm with and without the extended model.

When all topologies have been tested or scored, the algorithm returns all solutions of minimum cost. This process is described more precisely in Figure 15.

Our model makes no distinction between an event of zero cost and no event at all. Yet we would like to see only solutions that have the lowest number of events, so our algorithm uses (a version of) the number of events as a secondary criterion to rank the optimal solutions. For each tree in a given solution, we sum over all leaves the exons that are present in at least two leaves, and then divide this value by the number of exons that are present in at least one leaf and by the number of leaves. The result is an index between 0 (all exons are unique to their leaves) and 1 (all leaves have the same exons).

### Generating topologies

Topologies are generated with increasing numbers of trees. For each topology with *t *trees (*t*-topology), any edge removal yields a new topology with *t*+1 trees. However, that process alone does not suffice to generate all (*t *+ 1)-topologies. Therefore, once that procedure has been applied to all *t*-topologies (and duplicates have been removed), we use branch-swapping to generate the remaining (*t *+ 1)-topologies. A branch swap disconnects edge (*n*_1_, *p*_1_) from tree *t*_1_ and edge (*n*_2_, *p*_2_) from tree *t*_2_ and creates two new edges: (*n*_1_, *p*_2_) and (*n*_2_, *p*_1_). Here *n*_1_ and *n*_2_, and also *p*_1_ and *p*_2_, represent transcripts from the same ancestral gene. The algorithm again checks for duplicates, as it searches for all branch swaps on the set of (*t *+ 1)-topologies until no new topology can be generated.

### Scoring solutions and topologies

The score of a particular solution is composed of two parts, the first reflecting the structure of the trees and the second, *S_F_*, providing the parsimony score of the trees. The cost of creating or losing a transcript is a constant and thus we have

(1) $$S=\left(c_{B}\cdot N_{tree}+c_{D}\cdot N_{death}\right)+S_{F}$$

where *N_tree_* is the number of trees, *N_death_* the total number of transcript losses, and *S_F _*the sum of the maximum parsimony scores of each tree. *S_F _*is the only quantity that depends on the evolution of the gene's exons. *c_B_* and *c_D_* are parameters that control the cost of transcript birth and death.

A lower bound for topologies can be computed by considering the first part of the scoring function, *c_B_*· *N_tree_*+ *c_D_*· *N_death_*. This value does not depend on the transcripts, but only on the topology. However, a better lower bound can be computed by adding a lower bound on the *S_F_*score. For each tree, we compute the best leaf assignment as if all transcripts were available, corresponding to a topology with a single nontrivial tree. (In the real leaf assignment procedure, of course, trees compete for transcripts.) The sum of these values is a true lower bound.

### Leaf assignment procedure

Given a topology, leaf assignment remains challenging: given *N *genes and *k *transcripts per gene, a topology can lead to *k*!*^N-^*^1 ^possible leaf assignments. To tackle this problem, we combine a bottom-up dynamic programming algorithm with Sankoff's algorithm for the small parsimony problem.

We define a *state *as an ordered list of transcripts for a given gene. Each transcript *t *in a state has pointers to its left and right children, *l*(*t*) and *r*(*t*)--if any. The ordering of the transcripts is the same in two states of the same gene, but the pointers change to reflect phylogenetic relationships. The number of transcripts of ancestral genes (inner nodes) is defined by the topology.

For each ancestral gene, every possible state is generated. If a gene has *k_LR_* transcripts that have two children, *k_L _*transcripts with a single left child, *k_R _*transcripts with a single right child, and both children of the gene have *n *transcripts, then we have up to

(2) $$\left(\begin{array}{c} n\\ k_{LR} \end{array}\right)\cdot \frac{n!}{\left(n-k_{LR}\right)!}\cdot \left(\begin{array}{c} n-k_{LR}\\ k_{L} \end{array}\right)\cdot \left(\begin{array}{c} n-k_{LR}\\ k_{R} \end{array}\right)$$

possible states. The product of the first two terms of Equation 2 is the number of possible assignments for the *k_LR_* transcripts that have two children, while the last two terms compute the number of assignments for the transcripts that have only child. Since the first part selected *k_LR_* elements, there remains only *n *- *k_LR_* elements to choose from.

**Figure 16:**
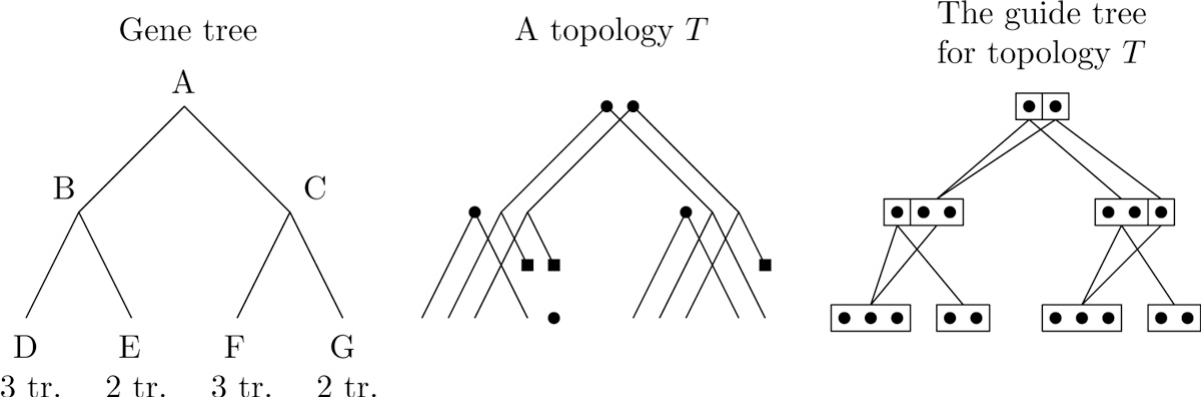
**The guide tree for a given topology**. **Topology**: There are two ancestral transcripts at *A*. Another transcript is created at *B*, two are lost between B and *E*, and a new transcript is created at *E*. A similar reasoning applies to *C*, *F*, and *G*. **Guide tree**: Boxes in the guide tree indicate a specific subtree. For instance, at *B*, we have only two kinds of subtrees: *B*(*D*,*E*) and *B*(*D*,-), thus we have two boxes. Within a box, each dot represents a transcript. At *B *we have two transcripts with subtree *B*(*D*,-), thus there are two dots in the second box.

However, this number is constrained by the topology: a transcript in some state cannot be connected to any transcript in its child's state--the subtrees have to match. We represent these constraints through a guide tree, which indicates the possible interactions for each transcript. Figure 16 illustrates the guide tree for an example topology. There could be up to 18 states at node *A *without the topology constraints, but these reduce the number down to just 4.

Our algorithm traverses the gene tree in postorder; at each node *N*, it computes the parsimony score of each state. Given a gene *N*, its parent *P*, and its two children *L *and *R*, the score for a given state *s *of *N *is given by

(3) $$\begin{array}{llll} S\left(s_{N},s_{P}\right) & =\underset{s_{R}\in R,s_{L}\in L}{\text{min}}\{S\left(s_{R},s_{N}\right)+S\left(s_{L},s_{N}\right)\; & & \;\\ & +\underset{t\in T}{\sum }\text{min}\text{MP}\left(t\right)\;\;\text{s}\text{.t}.\;T=roots\left(s_{N},s_{P}\right)\}\; & & \;\\ & \; & \end{array}$$

Where roots (*s_A_*, *s_parent_*) contains all transcripts of *s_A _*that have no ancestor in *s_parent_*. (If *s_parent _*is null then it is the set of transcripts in *s_A_*.)

minMP (*t*) is the parsimony score of transcript *t *as inferred by Sankoff's algorithm. A profile is built for each exon and the score of exon *i *in state *u *is computed by

$$t_{iu}=\underset{x\in E}{\text{min}}\left\{c\left(u,x\right)+r_{ix}\right\}+\underset{y\in E}{\text{min}}\left\{c\left(u,y\right)+l_{iy}\right\}$$

where *l *and *r *are the left and right children of transcript *t*, *c*(*a*, *b*) is the cost of evolving from *a *to *b *and *E *is the set of all exon states. In our case we have *c*(*a*, *b*) = 0 for *a *= *b *and *c*(*a*, *b*) = 1 otherwise. However the cost function must be slightly modified to account for exon evolution at the gene level. If a constitutive exon was gained or an exon was lost (at the gene level), then we set the cost of the change to zero. Additionally, if exon *i *is absent in the gene, then for all transcripts in the gene we have *t_ix_*= ∞,∀*x *> 0. Note that the left and right children of *t *depend on the choice of *s_L_*and *s_R_*. A similar equation can be derived in case of single-child transcripts. minMP(*t*) is then the sum over all exons of min*_u_t_iu_*.

The values in Equation (3) are assigned during the postorder traversal; once the score of every state at the root of the gene tree has been computed, the minimum score is retained. Backtracking from the root to the leaves will then produce all optimal transcript phylogenies.

### An extended model

The extended model sets a dynamic cost for transcript birth, but retains a constant cost for transcript death. Given a topology, the best leaf assignment is computed and backtracking is used to reconstruct the ancestral transcripts' sequences. Creation of new transcripts is assigned a cost that corresponds to its evolution from its closest ancestor. The birth cost is added only once the leaf assignment procedure has terminated and thus has no influence on the transcript assignment, except in case of multiple solutions, where only those that have a minimum birth cost will be selected.

Developing a good lower bound on the birth cost remains a challenge. This cost can vary between zero and the number of exons, so that simply using the lowest possible value would produce very loose bounds and thus be of no help in the search. (On simulated data and our two test genes, the search procedure using a zero value as a bound on the birth cost always had to look at every topology.)

## Conclusion

In this study we addressed the lack of evolutionary model for alternative splicing by presenting a two-level model of transcript evolution and an algorithm to reconstruct transcript phylogenies. Our work opens the door for the study of transcript evolution, as it provides tools for testing evolutionary hypotheses.

We presented two models. The basic model uses a fixed cost for the creation of new transcripts--an unrealistic assumption, but one that greatly decreases the computational cost. The extended model assigns a cost dynamically by finding the closest (least cost) ancestor; however, the dynamic nature of the cost defeats our pruning strategy and the problem became intractable for medium-sized instances.

Results on a well-studied gene, MAG, showed that the extended model yielded results similar to those obtained with the basic model. Good clustering of known isoforms was achieved with the basic model for both gene families (MAG and PAX6) we studied.

Future work involves a faster version of the algorithm, and eventually approximation methods to enable us to use the extended model on large problems.

## Competing interests

The authors declare that they have no competing interests.

## Authors' contributions

YC designed and implemented the algorithm, ran the different experiments, and drafted the paper; BMEM provided guidance and advice; both authors worked closely on the final draft.
